# Protein structure based prediction of catalytic residues

**DOI:** 10.1186/1471-2105-14-63

**Published:** 2013-02-22

**Authors:** J Eduardo Fajardo, Andras Fiser

**Affiliations:** 1 Department of Systems and Computational Biology, Albert Einstein College of Medicine, 1300 Morris Park Avenue, Bronx, NY 10461, USA; 2 Department of Biochemistry, Albert Einstein College of Medicine, Bronx, USA

**Keywords:** Functional site, Catalytic residues, Neural network, Feature selection, Structural genomics

## Abstract

**Background:**

Worldwide structural genomics projects continue to release new protein structures at an unprecedented pace, so far nearly 6000, but only about 60% of these proteins have any sort of functional annotation.

**Results:**

We explored a range of features that can be used for the prediction of functional residues given a known three-dimensional structure. These features include various centrality measures of nodes in graphs of interacting residues: closeness, betweenness and page-rank centrality. We also analyzed the distance of functional amino acids to the general center of mass (GCM) of the structure, relative solvent accessibility (RSA), and the use of relative entropy as a measure of sequence conservation. From the selected features, neural networks were trained to identify catalytic residues. We found that using distance to the GCM together with amino acid type provide a good discriminant function, when combined independently with sequence conservation. Using an independent test set of 29 annotated protein structures, the method returned 411 of the initial 9262 residues as the most likely to be involved in function. The output 411 residues contain 70 of the annotated 111 catalytic residues. This represents an approximately 14-fold enrichment of catalytic residues on the entire input set (corresponding to a sensitivity of 63% and a precision of 17%), a performance competitive with that of other state-of-the-art methods.

**Conclusions:**

We found that several of the graph based measures utilize the same underlying feature of protein structures, which can be simply and more effectively captured with the distance to GCM definition. This also has the added the advantage of simplicity and easy implementation. Meanwhile sequence conservation remains by far the most influential feature in identifying functional residues. We also found that due the rapid changes in size and composition of sequence databases, conservation calculations must be recalibrated for specific reference databases.

## Background

Worldwide structural genomics projects continue to release new protein structures at an unprecedented pace. To date nearly 6000 proteins were solved in the NIH-based Protein Structure Initiative, according to the weekly updated target tracking system Structural Biology Knowledgebase [1]. Stunningly, approximately 30% (1681/5736 as of April 21, 2012) of these new structures lack any type of functional characterization (http://sbkb.org/kb/search.do?type=unkstruc) let alone a specific description of residues critical for function. While sometimes the annotation of the fold that emerges from structural studies of a given target can provide a coarse functional classification [2,3], for the most part these structures do not have follow up functional studies. This presents a strong demand for computational methods that aim at identifying possible functional sites in these structural models. The most frequent and basic approach to functionally characterize proteins in general is to transfer functional annotation between proteins based on sequence similarity [4], typically after searching sequence databases with tools like Blast [5] or other sensitive, profile based search approaches [6,7]. While these methods can provide useful information, their applications are limited to proteins with high sequence similarity to other functionally characterized proteins and it is prone to, and partly responsible for, the propagation of errors in functional assignment [8]. The fraction of experimentally validated annotation represents only about 5% of all proteins according to benchmarks made on enzymes [9]. Estimates about the mis-annotation of enzymes (depending of the genome) varies between 5-40% [8,10]. Previous studies argued that sequence based approaches are reliable only if at least 40-50% sequence identity exists between two proteins. For example, in the case of enzymes, a similar (but not necessarily identical) function can be assumed between two proteins if their sequence identity is above 40%, but if the sequence identity drops in between 30-40% then only the first three Enzyme Commission (EC) numbers can be predicted reliably, and only at 90% accuracy level. Below 30% sequence identity, structural information is necessary to essential for functional annotation [11,12]. Meanwhile it is estimated that 75% of homologous enzymes share less than 30% identical positions [12]. Other surveys reported that less than 30% of enzymes that share more than 50% sequence identity will have identical EC numbers [11]. Given that the average sequence identity between structurally related proteins is ~8-9%, and most of these share less than 15% identity [13], we must expect a high degree of functional diversity in proteins with similar folds [14]. This indicates an imperative need for structures and structure based approaches for functional annotations of these proteins.

An alternative approach to functional annotation, which also provides a more detailed insight, is the identification of residues that are critical for protein function. The expected benefit is that the knowledge of residues important for protein function can serve as a guide to experimental approaches, such as mutational studies, to test, confirm, or manipulate function. Methods for identifying functional residues can be broadly divided into those based solely on sequence information, those that rely on structural information, and those that combine the two in their predictions. For sequence based methods, analysis of Multiple Sequence Alignments (MSA) is the most informative procedure. Typically, a MSA is constructed after searching a sequence database, and then each column of the MSA is evaluated and a conservation or an entropy score is assigned to the column. Scoring the MSA can be complemented with prediction of secondary structures, relative solvent accessibility, or catalytic propensity of amino acid types. For instance, CRpred [15] uses the Position Specific Substitution Matrix and entropy values extracted from the MSA, hydrophobicity values calculated in a variable-sized window, and the separation between catalytic residue pairs in the protein sequence for training a Support Vector Machine. Another sequence-based method, FRcons [16], calculates a score related to the relative entropy, and uses the predicted solvent accessibility and secondary structure to estimate the background distribution of each residue. A class of sequence based methods use a MSA and a phylogenetic tree for a protein family [17-19]. The tree is analyzed from the root to the leaves searching for patterns of conservation at each node, looking to define residues important for function of the entire family or residues that are specific for each subfamily. If a structure is known, the residues identified can be further mapped onto the surface of the protein. Structure based methods are very diverse in their approaches. A variety of methods were developed that perform an unbiased search (i.e. not using any template library of functional motifs) for recurring structural motifs [20-22]. These methods are powerful when similar functionalities exist in different folds, or when function is explored in a structurally divergent Superfamily. One unbiased structure based technique that has been repeatedly used in various applications relies on graph theory. Using the atomic coordinates of the amino acid residues, pairs of interacting residues are identified and the information of a protein structure is converted into a network of interacting residues. The residue pairs are used to build the edges of a graph, where the nodes are the residues and the edges represent interactions. Once a graph is made, one can score the relative importance of each residue (node) in the protein (graph) by calculating a variety of possible centrality measures, which are often assumed to be good predictors of functional residues. The underlying assumption in these approaches is that functional residues display a pattern of connectivity to the rest of the protein that ranks higher than the pattern of non-functional residues. While most of the methods that use a graph approach complement the centrality measures with sequence based scores (see below), SARIG [23] finds pairs of interacting residues with the CSU program [24], and predicts catalytic residues based on closeness centrality values combined with relative solvent accessibility. Other features used for structure based prediction include the analysis of mechanical properties of each residue [25] because catalytic residues are assumed to be more difficult to move with respect to other residues in the protein. Other structure based approaches take into account the shape of the protein surface, looking for the largest clefts in the structure, where catalytic residues tend to reside [26,27] or the “deepest” yet still exposed residues [28]. In THEMATICS [29], a graph of proton occupation against pH is prepared for each ionizable residue; functional residues display distortions in these graphs, and are identified by having at least one other such residue in the vicinity.

Methods that rely exclusively on sequence information are valuable when no structural information is available. However, when the structure of a protein is known, the best performing methods make use of both sequence and structural information. Sequence conservation is probably the most powerful attribute for identification of functional residues, and some flavor of sequence conservation analysis is present in virtually all these hybrid methods. Thiebert et al. [30] build a graph of interacting residues and calculate the *degree*, *degree-2*, clustering coefficient and closeness for each node, and conclude that the best predictor relies on closeness centrality and phylogenetic analysis. Here, the *degree* of a node is the number of nodes directly connected to it, that is, nodes that are exactly one edge apart. The *degree-n* of a node is the number of nodes that are exactly *n* edges apart. A related approach uses *degree*, *degree-3* and residue type combined with the propensity of each residue type to be catalytic, which is calculated as a percentage over their database of catalytic residues [31]. Petrova et al. uses sequence conservation, catalytic propensity of amino acids, solvent accessibility and relative position to the clefts of the structure [32], while Cilia and Passerini combines these sequence based measures with other physicochemical traits of structural neighborhoods, defined as those residues contained in a spherical region of 8 angstrom centered on each amino acid in the structure [33]. The Partial Order Optimum Likelihood (POOL) method [34], improves on the analysis of the structure-based THEMATICS method, by extending it to non-ionizable residues and combining it with sequence conservation and cleft size information using a SVM.

In this paper, for the purpose of feature selection, we explore the pairwise correlation between some of the attributes most frequently used for the prediction of functional residues, namely, the centrality measures of closeness, betweenness and page-rank, in addition to distance to the general center of mass (GCM) [35], relative solvent accessibility (RSA) and sequence conservation. We found that several of the graph based features strongly correlate with one another and with the distance to GCM but capture only partially the signal of functional residues. We explore possible combinations of these inputs for training neural networks, and identify a simple set of factors for efficient selection of catalytic residues based on sequence and structural information. We also explored the effect of the rapidly changing reference databases on the accuracy of residue conservation calculations and observed a strong dependence, which suggest that approaches that include this feature must be either regularly recalibrated or a specific reference database must be designated.

## Results and Discussion

### Feature selection to predict functional residues

Methods for the identification of functional residues rely on a wide variety of attributes that differentiate functional and non-functional amino acids. These features include sequence conservation, closeness, betweenness, RSA, distance to the GCM, and many others. Some of these measures are related to one another, which means that some seemingly different methods are in fact analyzing the same underlying property. Therefore, we first analyzed the 439 structures in the training dataset (see Materials and Methods) and scored every residue for each of the attributes included in this study. Then, we calculated the cross correlations between variables used, to shed light on the relatedness among existing methods for predicting functional residues (Table 1.) In the remainder of this paper, when we discuss functional residues, we will be referring to those annotated as catalytic in the CSA database [36], unless otherwise noted in the context.

**Table 1:** Correlations between all pairs of variables considered in this study


**Distance**	−0.2949					
**Closeness**	0.4026	−0.7765				
**Between**	0.3504	−0.4898	0.7452			
**PageRank**	0.3163	−0.2732	0.4977	0.8068		
**RSA**	−0.3577	0.4067	−0.5901	−0.6053	−0.7136	
**Function**	0.1546	−0.1340	0.1363	0.1086	0.0594	−0.0458
	**Conserv**	**Distance**	**Closeness**	**Between**	**PageRank**	**RSA**

Conserv: sequence conservation; Distance: distance to general center of mass; Closeness: closeness; Between: betweenness; PageRank: PageRank, RSA: relative surface accessibility.

From the results (Table 1), it is immediately clear that sequence conservation shows the highest correlation with function, as has been suggested in several previous studies [15,32,37]. Interestingly, function also shows a relatively high absolute correlation with distance to GCM and closeness, followed by betweenness. Perhaps not surprisingly, distance of an amino acid residue to the GCM has a high negative correlation with closeness, a reflection of the fact that residues near the center of the protein are relatively close to all other residues in the protein, and this is captured in the graph of interacting residues. Likewise, other node centrality measures vary negatively with the distance of the residue to the GCM. All the node centrality measures are highly correlated to one another, indicating that in these graphs, a residue with a high centrality value according to one measure is likely to have high centrality according to another measure. We used these insights in our exploration of the optimal set of features used as inputs to neural networks prediction for the identification of functional residues.

### Correlation of functional and non-functional residues with all the features studied

**Figure 1:**
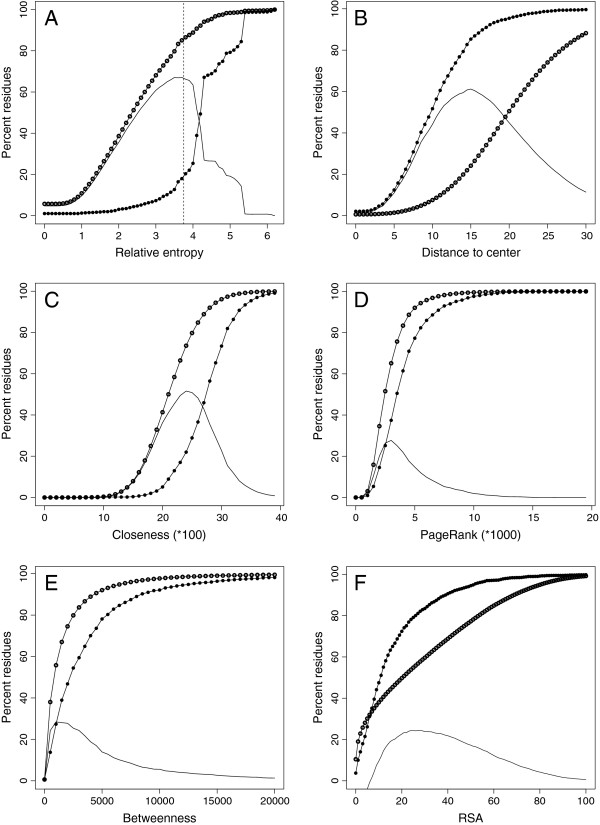
**Cumulative distribution of functional (filled circles) and non-functional (open circles) residues for each of the attributes analyzed.** The plain solid line shows the relative difference between the two other lines. **A**, sequence conservation using relative entropy; dotted line indicates the threshold value used for selection of residues before presenting to neural networks; **B**, distance to the GCM; **C**, closeness; **D**, PageRank; **E**, betweenness; and **F**, Relative Surface Accessibility.

The coarse analysis with the Pearson’s correlation coefficient between function and each of the features studied (Table 1) suggests that it is possible to separate functional and non-functional residues based on each of the attributes analyzed, perhaps with the exception of RSA, which shows almost no correlation. To visualize this segregation, we plotted the cumulative distributions of functional and non-functional residues as a function of each variable. There is a clear separation between the two curves characterizing functional and non-functional residues for all the attributes studied (Figure 1). The greatest discrimination between functional and non-functional residues is achieved with conservation analysis (Figure 1A), followed by distance to the GCM (Figure 1B). Closeness also gives good separation but then the curves are closer to each other for the remaining variables. Separation of the curves, therefore, is in agreement with the global correlation values, except for RSA, where the curves are well separated starting at approximately 10% RSA but they cross each other for lower RSA values. In this training set, approximately 30% of the functional residues have RSA of 5% or less, and approximately 10% are completely buried. This is consistent with previous reports on the overall accessibility of functional residues, and explains the low global correlation of RSA and function [38].

### Neural networks

Based on the observations described in the previous section, we set out to define a function for identification of functional residues, one that combines some or all of the features selected and returns a fraction of the input residues that is highly enriched in functional residues. According to the cross-correlation table, the use of some of the inputs is redundant and adds little to the overall result. Since it is likely that the features have to be combined non-linearly for functional residue identification, we trained neural networks to integrate the different inputs into a single function. We used the information on the cross-correlation table as a guide to our exploration of the set of attributes presented to the neural networks. A set of 89 structures was randomly selected from the initial training set of 439 structures, and used as a validation set to make comparisons between the different training runs. The training of the neural network was done on the remaining 350 structures of the original training set (see Methods). Based on our results (Figure 1A and Table 1) and on previous reports that conservation is the most useful feature for identifying functional residues, we initially trained networks either with the entire set of residues or only with conserved residues, those with a relative entropy measure of 3.5 or larger. Residues were presented to the networks and analyzed with different combinations of measures for the attributes in Table 1. The best performing networks were those that used distance to the GCM and amino acid type as inputs (Table 2). Adding any other input always resulted in a reduced performance, as monitored with the Matthew's Correlation Coefficient (MCC) on the validation set (see Methods). Table 2 shows that preselection of conserved residues always resulted in performance improvements with any combinations of inputs. Consequently, we decided to optimize the step of preselecting residues based on sequence conservation, and used distance to the GCM and residue type as inputs to the neural networks. We retained the weights for the neural connections of the best performing network for further evaluation. Figure 1A shows how selection of an appropriate sequence conservation level can increase the proportion of functional residues in the input set, by eliminating large numbers of non-catalytic residues, while retaining most of the catalytic ones. For example, elimination of residues with relative entropy of 3.5 or less removes 79.8% of non-functional residues but only 12.7% of functional residues. After this trade-off step we retain 1,519 out of 1,740 functional residues and 30,699 of 158,458 non-functional residues of the entire training set, resulting in a set that contains approximately 5% functional residues, a nearly 5-fold enrichment over the original input. Also, at a relative entropy value of 3.5, the slope of the functional residue curve shows a dramatic increase, with a bigger increase at 4.0 (Figure 1A). A comparison with the slope of the non-functional residue curve indicates that beyond some point near 3.5 we start discarding functional residues at a greater rate than non-functional residues, and this is reflected in the downward trend of the plain solid line in Figure 1A. To confirm this visual observation, we preselected residues from the validation set using relative entropy values between 2.5 and 4.0 in increments of 0.1, and used each enriched set as input to the best performing network trained with residues that show entropy values of 3.5 or better and with distance to GCM and residue type as the only attributes. The calculated MCC after feeding the various enriched sets to the neural network peaked when we discarded residues with a relative entropy value of less than 3.8; at this level, we retain 1378 functional and 20266 non-functional residues, producing a set that contains 6.8% functional residues. We selected this value of 3.8 for use in the final protocol, and to measure performance with the independent testing set of 29 structures.

**Table 2:** A representative list of performances of neural networks with different combinations of features used as inputs

**Feature**				**Matthew’s correlation (%)**	
C	D	A	K	All residues	Conserved > 3.5
X				16.28	22.05
	X			15.51	26.68
		X		10.12	27.56
X		X		18.71	28.16
	X	X		19.78	32.48
X	X			20.56	27.67
X	X	X		20.68	28.95
		X	X	10.95	25.48

C, sequence conservation; D, distance to the GCM; A, residue type; K, closeness. A check symbol on a column indicates that the feature was included in training. The Matthew’s correlation coefficient, expressed as a percentage, was calculated on the validation set after convergence of the training procedure. Networks were trained either with “All residues” or only with residues that showed a conservation value of 3.5 or more. The calculation of the MCC was performed over the entire original set of residues, regardless of the effect of preselection on the overall counts.

### Comparison with existing methods

There is a paucity of publicly available methods for the prediction of functional residues based on protein structures. As a result, it is difficult to do a fair comparison of a newly developed method with other existing methods; therefore, we relied on the performance reports in the original publications. However, one should note that these performances were tested on different datasets and therefore cannot be compared with one another directly. The reportedly better performing methods [33,34] appear to be demanding in terms of computational power, which also could be a possible explanation for their limited availability to the public. For instance, POOL [34], which supplements the proton occupation prediction of THEMATHICS with surface cleft and sequence conservation analysis, is one of the methods that report higher performance, with 64.7% sensitivity and 19.07% precision, for a 29.46 F1-measure value. Using a 10-fold cross-validation protocol, Cilia and Passerini [33] present a high 28% precision value at the expense of a relatively low 46% sensitivity, resulting in a 34.81 value for the F1-measure. The method of Cilia and Passerini makes use of sequence information and the composition of structural neighborhoods. On the other hand, Petrova and Wu [32], using sequence conservation, solvent accessibility and position relative to surface clefts, achieve high sensitivity (89.8%) but low precision (6.98), producing a 12.95 F1-measure score. Our testing set consisted of 29 structures that were not used during the development process, containing 111 residues that were annotated as catalytic in Catalytic Site Atlas out of 9262 total, or 1.2% functional residues. After applying the entire procedure, we selected 411 (4.4%) of the input, which contained 70 of the catalytic residues (63.06%). Therefore, annotated catalytic residues make up 17.03% of our output, representing an enrichment of over 14-fold when compared to the input set. To obtain a more robust measure of performance, we carried out a 10-fold cross-validation procedure on the entire dataset, after clustering at 30% sequence identity. The average sensitivity after cross-validation was 60.14%, with an average precision of 18.26% and an F1-measure score of 28.01 (see Methods).

Table 3 shows how our figures compare to those from other methods of functional residue prediction. The only working server for structure based functional residue prediction that we found was that of SARIG [23], and we submitted our 29 structures for analysis. SARIG uses closeness centrality and RSA to predict catalytic residues. Our method clearly outperforms the structure-only SARIG (Table 3A). It was also of interest to make a comparison with a sequence based prediction method, so we contacted the authors of CRpred [15], perhaps the best sequence-only method available, who graciously agreed to analyze our testing set. Compared to CRpred, our method has better sensitivity (63.06% vs 51.35%) but lower precision (17.06% vs 21.75%), resulting in a lower F1-measure (26.82 vs 30.56). This was confirmed with a different dataset, the EF-fold set, originally used by Youn et al. [39] and later evaluated by CRpred. Table 3B shows the results of a 10-fold cross-validation analysis of the EF-fold dataset. Our method achieved higher sensitivity than both CRpred and the method of Youn et al. but lower precision and F1-measure. Youn et al. use the structural conservation score of S-BLEST [40] together with sequence conservation and residue hydrophobicity as inputs to a Support Vector Machine [39]. In the 10-fold cross-validation procedure, our method achieved higher sensitivity than CRpred (55.7% vs 48.2%) and lower precision (14.09% vs 17.0%), with F1-measures of 22.49 and 25.13, respectively. Thus, our method is better at identifying functional residues (resulting in higher sensitivity) but CRpred recognizes, and discards, non-functional residues at a higher rate (achieving higher precision), as does the method of Youn et al. (51.1% sensitivity, 17.13% precision). This difference might be explained, at least in part, by the makeup of the training sets: for the balanced training of the neural network we used a 1:1 ratio of functional:non-functional residues, while CRpred and Youn et al. used at 1:6 ratio in their Support Vector Machine based method. In any case, the performance of CRpred is especially remarkable since it uses no structural information. In the next section we briefly explore one major aspect that can influence the results of sequence profile based analysis: the rapidly changing sequence databases.

**Table 3:** Performance of selected catalytic-residue prediction methods

**A**			
	**Sensitivity**	**Precision**	**F-Measure**
**Conservation-distance-aa**	63.06	17.06	26.82
**Sarig-server (2004)**	54.05	7.85	13.71
**CRpred (2008)**	51.35	21.75	30.56
**B**			
**Conservation-distance-aa**	55.7	14.1	22.49
**CRpred (2008)**	48.2	17.0	25.13
**Youn et al. (2007)**	51.1	17.13	25.66

A. Comparison of three methods using our independent testing set of 29 proteins. B. Comparison of three methods based on a 10-fold cross-validation procedure on a common dataset.

### Effect of rapidly growing databases

Sequence conservation is the most influential trait in many functional residue prediction protocols, including the one presented here. A necessary step in finding a conservation value of an amino acid residue is the comparison of a query sequence with all the sequences in an all-inclusive reference database. One obvious consequence of such comparison is that conservation values are sensitive to the size and redundancy of the selected reference database. This is of particular relevance given the exponential growth of sequence databases, and leads to the question of whether their overall information content is approaching some sort of saturation point. This would imply the unlikely condition that all types of sequence information entering the database provide only more redundancy. From a practical standpoint, a perceived saturation is necessarily tied to a query sequence, with some query sequences having hundreds or thousand of relatives, while others display few or none, depending on the parameters used in the search. Thus, the overall performance measure of a method that relies on sequence conservation is affected by changes in both the specialized test database and in the reference sequence database, which are, for the method presented in this paper, the CSA and the non-redundant protein database of NCBI (nr), respectively.

An examination of the growth of CSA, from its initial release in 2004 [36] through the latest release in 2010, indicates that the number of non-redundant protein chains with annotated catalytic residues extracted from the literature (as opposed to those inferred by sequence similarity) has increased from 714 to 913 or 28% over 6 years. The number of annotated catalytic residues in those releases has grown proportionally, from 2235 to 2948, for a 32% increase, indicating that there has been no dramatic change in the annotations. During the same period, nr has grown from about 2 million to 12 million sequences, which is more than a 600% increase. Based on those raw numbers, any database-dependent change in the performance of a method over these years is likely due to the changes in nr, with the growth of CSA having a modest effect. The relative growth of nr is magnified because CSA is a relatively diverse database: for instance, clustering at 50% sequence identity decreases the effective size of CSA by only approximately 5%. In contrast, the internal redundancy of nr is much greater. While measuring the size reduction of nr at different levels of sequence identity is computationally intensive, clustering at 50% sequence identity produces a 70% reduction in size in Uniref [41], and we expect a similar behavior for nr. This implies that the growth of nr comes largely at the expense of adding redundant information, in comparison to CSA.

The analysis presented throughout this paper was done using a release of nr of March 2010 as our reference database, when the size of the database was approximately 12 million sequences. The current release of nr, in September of 2012, already has approximately 17 million sequences, an additional growth of about 40% over the 2010 release. To determine how the use of this new release affects our numbers, we recalculated our sequence conservation values with the current nr release, and found that using the test dataset of 29 proteins, the sensitivity value stayed the same at 63.03% but the precision dropped from 17.03% (Table 3) to 14.40%, resulting in an F1-measure score of 23.45. The drop in precision was due to more residues meeting the minimum conservation value requirement of 3.8, probably, as observed above, because more homologous sequences entered the database. Therefore, we proceeded to identify a new optimal threshold value of 4.0 for this increased and more redundant version of nr using the original testing dataset. With this new threshold, we obtained a sensitivity of 60.36% and precision of 15.95%, for an F1-measure value of 25.23. Thus, with the new database and the new threshold, we almost recapitulated some of the original performance figures of the 2010 analysis. This exercise sheds light on the dynamic interplay between rapidly evolving and changing sequence databases and reveals the need for recalibration of parameters as the public databases change. This also highlights a complication in comparing performances of different methods over the years. Sequence conservation based approaches either have to use a frozen reference database or need to be regularly updated and recalibrated.

### Illustration of predictions of functional residues

**Figure 2:**
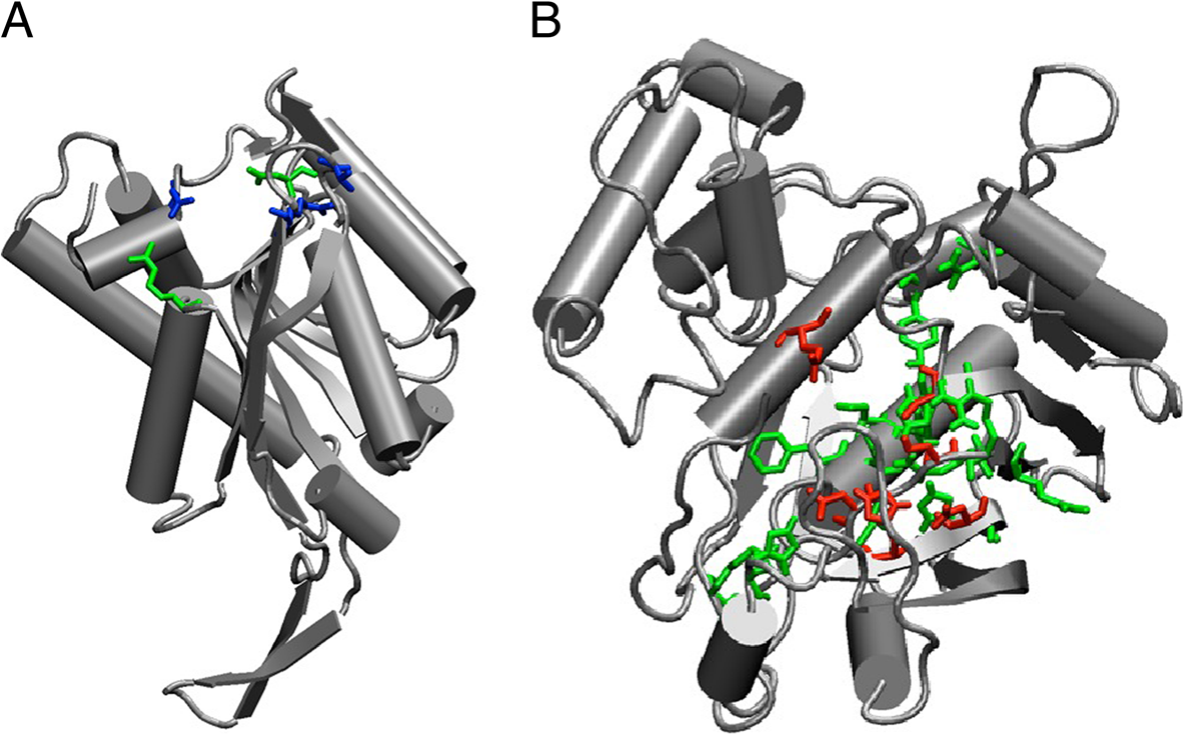
**Predicted (green) and experimentally characterized (red and blue) functional residues.** Experimentally characterized functional residues that were correctly predicted are marked in red while those that were missed are in blue. (**A**) one case where several methods failed (1bd3) (**B**) a successful case (all six functional residues captured) 1lcb.

The results presented in the previous section are the aggregate figures over the entire testing set. Although useful for the evaluation of the method as a whole, those figures provide little information on the specifics of each structure analyzed. In particular, the method failed to find any of the catalytic residues in six of the 50 structures of an expanded version of the testing set. Meanwhile, it found all the annotated catalytic residues in 11 of the structures. As a comparison, SARIG found no functional residues in 3 structures and all residues in 6. Both methods failed for one structure, 1BD3, which corresponds to a *Toxoplasma gondii* uracil phosphoribosyltransferase (Figure 2A). Five of the six structures for which our method failed, including 1BD3, illustrate those cases where high sequence conservation is not seen in catalytic residues; however, there are conserved residues in those structures that are not annotated as catalytic. As shown in Figure 1A, approximately 18% of the functional residues are eliminated at our required level of conservation. Also, Table 1 shows that residue conservation correlates with the distance to the center of mass. This is in accordance with previous reports, which observed that in addition to catalytic residues, those that are buried at the “core” of a structure show a rate of mutation lower than other residues in the structure [10]. Residues that make the core are often responsible for the structural integrity of the protein so it is not surprising that they tend to be conserved; from this perspective, core residues could be regarded as essential for protein function since catalytic residues depend on a specific spatial distribution to act efficiently on a substrate [42]. Because of the specifications of its design, our method is likely to return residues of the core.

In a successful case examined, the structure 1LCB of thymidylate synthase from Lactobacillus casei thymidilate synthase, our method selected 20 of the original 315 residues or 6.3% of the structure (Figure 2B). The output contained all the 6 annotated catalytic residues for a 30% precision.

## Conclusions

Annotation of catalytic residues is an arduous task that is prone to errors. We limited our dataset to annotations for which there is experimental evidence reported in the literature, leaving aside annotations based on sequence similarity. Still, those annotations are probably not perfect as illustrated by the case of 1B3R, for which slightly different sets of catalytic residues are reported in CSA, which served as our source of data, and in the related Catalytic Residue Set (CATRES) [43], with histidine 54 found in CSA but not CATRES. Thus, some of the residues classified as false positives might in fact be involved in catalysis but not identified as such in CSA.

Prediction of functional residues is inherently difficult due to the often poor understanding of which residues play a critical role in protein function, in addition to those involved in a direct chemical reaction. It is assumed that residues responsible for catalysis evolve very slowly as catalytic capability imposes the strongest constraint on the fitness of an enzyme [10]. Meanwhile, residues that are involved in substrate selectivity (binding) have more flexibility, partly due to the variety of compensatory mutations that can establish a similar environment [44]. Yet, other structurally remote residues, while not directly involved in the reaction or selectivity, might be critical for function by playing role in “promotional” vibrations for efficient catalytic reaction [45] or fulfilling structural roles essential for activity [10,46]. Methods that identify functional residues in fact may contribute to uncovering the network of residues responsible for function and to a better understanding of the role of the entire structure [47].

## Methods

### Testing and training sets

Version 2.2.11 of the Catalytic Site Atlas database [36] was downloaded (http://www.ebi.ac.uk/thornton-srv/databases/CSA_NEW/). PDB chains were collected for entries where the evidence for functional residues comes from the literature. Further, only those chains with a single Site identifier and between three and twelve catalytic residues were retained, and proteins with multiple annotated active sites were not considered. We also eliminated 12 proteins that were 120 residues or shorter. Finally, we clustered with CD-hit [48] at 60% sequence identity, and obtained a non-redundant set of 525 protein chains. After removing entries where some annotated residue positions are not amino acids, we were left with 489 chains. A random sample of 50 proteins was selected for testing; the remaining 439 chains were used for analysis and training purposes. The training set contains 158,458 residues of which 1740 (1.1%) were annotated as functional, and the rest assigned as non-functional. We also explored performance on an even less redundant testing set at 30% sequence identity. This reduced the size of the testing set to 41. For direct performance comparison with CRpred [15], we downloaded the EF-fold dataset used for training of the CRpred model (http://biomine.ece.ualberta.ca/CRpred/CRpred.htm) and removed any proteins that are within 30% sequence identity to any protein in the EF-fold dataset. We were left with 29 proteins that make up our independent testing set.

For the cross-validation procedure, the entire 489-protein set described above was clustered at 30% sequence identity with CD-hit. The resulting 437 sequences were divided randomly into 10 similarly sized sets.

### Features

A table of interacting residues was prepared for each PDB chain using the Contacts of Structural Units (CSU) program [24]. Each residue pair in a predicted binary interaction was then used to build the edges of a graph, using the Graph::Undirected module of Perl. The distance between each node of an interacting pair, i.e., the length of an edge, was set at 1. To determine the relative importance of each amino acid in the protein, we calculated the closeness centrality, betweenness centrality and page-rank centrality of each node. Closeness of a node is defined as the inverse of the average geodesic distance (or length of the shortest path) to all other nodes; betweenness indicates how many of the shortest paths between any two nodes in the graph include the subject node; and page-rank centrality is a measure developed for searching web pages in the internet, related to eigenvalue centrality, where an important node is one whose neighbors have many connections to other nodes [49]. For the prediction of functional residues, rather than working with raw centrality values, we calculated the rank percentile of each node for each centrality measure and expressed it as a fraction, so that each node is assigned a value in the interval (0,1]. Closeness and betweenness centrality values were calculated with the Algorithm::SocialNetwork Perl module; page-rank centrality was calculated using the Graph::Centrality module.

The coordinates of the General Center of Mass (GCM) of a protein were determined using the coordinates of the heavy atoms of each residue. For each *x*, *y* and *z* coordinate, the position of the GCM was calculated as $R=\sum_{i}\left(M_{i}*R_{i}\right)/\sum_{i}M_{i}$, where *M*_*i*_ and *R*_*i*_ are the mass and the corresponding coordinate of atom *i*. The distance of an amino acid to the GCM was defined as the distance between its Cα and the GCM.

Relative solvent accessibility (RSA) values were calculated using the naccess program [50]. Pearson’s cross correlations were determined with the R statistical package (http://www.r-project.org/).

For the residue conservation value calculation, the sequence of the protein was extracted from the ATOM records of the PDB file and compared with all the proteins in the non-redundant database of NCBI (NR) using three rounds of PsiBlast with an e-value of 0.001 [51]. A multiple sequence alignment was generated by consolidating the pairwise alignments of the PsiBlast output, using BlastProfiler [52]. To reduce redundancy among hits and to ensure high quality alignments, we required that each hit cover at least 75% of the query sequence (this is the BlastProfiler default) and that the maximum sequence identity between any two hits be at most 95%. We did not apply any additional weighting for each sequence, following the results of Johansson and Toh [53]. To assign a conservation value to each residue in the query sequence, we calculated the relative entropy as described in Wang and Samudrala [54].

### Performance measures

The primary measure that we used to monitor performance was the Matthew's Correlation Coefficient, defined as $\text{MCC}=\frac{\left(\text{TP×TN}\right)-\left(\text{FP×FN}\right)}{\sqrt{\left(\text{TP}+\text{FP}\right)\left(\text{TP}+\text{FN}\right)\left(\text{TN}+\text{FP}\right)\left(\text{TN}+\text{FN}\right)}}$, where TP, TN, FP and FN are the numbers of true positives, true negatives, false positives and false negatives, respectively. Other, more informative measures are used to present the data, including sensitivity (number of functional residues identified divided by the total number of annotated functional residues) and precision (number of functional residues identified divided by the number of residues returned). To facilitate comparisons with other existing methods, we also calculated the F1-measure, which incorporates the concepts of sensitivity and precision in a single number, and is defined as $F_{1}=2\times \frac{\text{Sensitivity}\times \text{Precision}}{\text{Sensitivity}+\text{Precision}}$

### Neural networks

Supervised, feed forward neural networks with one hidden layer of ten units were trained using the back propagation algorithm [55]. For training, a random sample of 89 structures from the training set was put aside as a verification set, and was used to select the best performing network. The remaining 350 structures were used for the actual training. Since the input is highly unbalanced, with about 100 times fewer functional residues, a random sample of non-functional residues was selected to present the network with the same number of functional and non-functional residues. The residue type was encoded as 20 separate inputs, one for each type. The input corresponding to the relevant residue was assigned a value of 1 and all other inputs were set to zero.

## Competing interests

The authors declare that they have no competing interests.

## Authors’ contribution

EF: concept, design and carried out the calculations, writing the manuscript; AF concept, design, writing the manuscript. Both authors read and approved the final manuscript.
